# Exploring Burnout among Nursing Students in Bangalore: A t-Distributed Stochastic Neighbor Embedding Analysis and Hierarchical Clustering in Cross-Sectional Data

**DOI:** 10.3390/nursrep14030126

**Published:** 2024-07-16

**Authors:** Michael Sebastian, Maddalena De Maria, Rosario Caruso, Gennaro Rocco, Cristina Di Pasquale, Arianna Magon, Gianluca Conte, Alessandro Stievano

**Affiliations:** 1 Department of Biomedicine and Prevention, University of Rome Tor Vergata, 00133 Rome, Italy; michael.sebastian@students.uniroma2.eu; 2 Department of Life Health Sciences and Health Professions, Link Campus University, 00133 Rome, Italy; maddalena.demaria@outlook.it; 3 Department of Biomedical Science for Health, University of Milan, 20133 Milan, Italy; 4 Clinical Research Service, IRCCS Policlinico San Donato, 20097 San Donato Milanese, Italy; 5 Faculty of Medicine and Surgery, University Our Lady of Good Counsel, 1001 Tirana, Albania; g.rocco@prof.unizkm.al; 6 Stomacare Service, European Institute of Oncology, IRCCS, 20141 Milan, Italy; 7 Health Professions Research and Development Unit, IRCCS Policlinico San Donato, 20097 San Donato Milanese, Italy; arianna.magon@grupposandonato.it (A.M.); gianluca.conte@grupposandonato.it (G.C.); 8 Centre of Excellence for Nursing Scholarship, 00136 Rome, Italy; astievano@unime.it; 9 Department of Clinical and Experimental Medicine, University of Messina, 98122 Messina, Italy

**Keywords:** burnout, nursing education, hierarchical clustering, t-SNE analysis, student well-being, India

## Abstract

This study explores burnout among nursing students in Bangalore, India, focusing on Exhaustion and Disengagement scores. A cross-sectional design was applied using the Oldenburg Burnout Inventory modified for nursing students, collecting data using a survey that was conducted between October and December 2023. The sample consisted of 237 female nursing students from the Bachelor of Science in Nursing program at Bangalore College of Nursing, South India. The study integrated the t-distributed Stochastic Neighbor Embedding (t-SNE) procedure for data simplification into three t-SNE components, used in a hierarchical clustering analysis, which identified distinct student profiles: “High-Intensity Study Group” and “Altruistic Aspirants”. While burnout scores were generally high, students with high study hours (“High-Intensity Study Group”) reported greater Exhaustion, with a mean score of 26.78 (SD = 5.26), compared to those in the “Altruistic Aspirants” group, who reported a mean score of 25.00 (SD = 4.48), demonstrating significant differences (*p*-value = 0.005). Conversely, those motivated by altruism (“Altruistic Aspirants”) showed higher Disengagement, with a mean score of 19.78 (SD = 5.08), in contrast to “High-Intensity Study Group”, which reported a lower mean of 17.84 (SD = 4.74) (*p*-value = 0.002). This segmentation suggests that burnout manifests differently depending on the students’ academic load and intrinsic motivations. This study underscores the need for targeted interventions that address specific factors characterizing the clusters and provide information for designing future research and interventions. This study was not registered.

## 1. Introduction

Burnout among healthcare professionals, especially nursing students, is increasingly recognized as critical, necessitating accurate measurement and early intervention [1,2,3,4,5]. Defined primarily as a state of physical, emotional, and mental exhaustion caused by prolonged stress, burnout severely impacts both personal well-being and professional performance [6]. This condition diminishes personal well-being and significantly impairs professional performance and patient care. A recent systematic review and meta-analysis reported a burnout prevalence of 19% among nursing students, with emotional exhaustion affecting up to 41% of students, highlighting the urgency of addressing this issue within educational and clinical settings to prevent its detrimental impacts on future healthcare services [1].

Burnout, historically recognized as a psychological phenomenon [7], has evolved into a critical topic in occupational health. This condition is typically described through a tri-factor structure proposed by Maslach and others, consisting of emotional exhaustion, depersonalization, and reduced personal accomplishment [7]. Emotional exhaustion is the core individual stress component, reflecting feelings of being emotionally overextended. Depersonalization represents a cynical or detached response to one’s work environment, the interpersonal component. In contrast, reduced personal accomplishment, which is inversely related to the other two dimensions, reflects a diminished sense of competence and productivity, capturing the self-evaluation component of burnout [6,7].

The COVID-19 pandemic has exacerbated these issues, leading to unprecedented levels of burnout among nurses due to increased workload, emotional strain, and the need for prolonged use of personal protective equipment. Studies have reported significant correlations between burnout and factors such as insufficient staffing, high patient load, and inadequate support, particularly during the pandemic [8]. For example, a literature review found that 68% of nurses met the criteria for the exhaustion dimension of burnout, and 88.3% met the criteria for the disengagement dimension during the COVID-19 pandemic [8]. Another study highlighted the urgent need for healthcare organizations to implement strategies to mitigate burnout, including mindfulness training, self-care techniques, and providing adequate rest and psychological support [9]. These findings sustain the necessity of addressing burnout comprehensively to ensure the well-being of nursing professionals and the quality of patient care.

In the last two decades, a two-dimensional perspective on burnout has gained traction in nursing education and practice, positing that disengagement and exhaustion are the fundamental dimensions of burnout, with reduced personal accomplishment playing a less pivotal role [10]. This perspective suggests that the connections between reduced personal accomplishment and burnout outcomes and antecedents are weaker than exhaustion and disengagement [11]. Furthermore, evidence indicates that while emotional exhaustion often leads to disengagement, reduced personal accomplishment evolves independently, acting more as an individual difference akin to self-efficacy rather than a core component of burnout [12]. This two-dimensional approach could be strategically advantageous in research and practice as it simplifies the model of burnout, focusing on the primary stressors that affect most individuals. This approach allows for more targeted interventions that directly address the most impactful elements of burnout, potentially enhancing the effectiveness of treatment and prevention strategies.

In this context, The Oldenburg Burnout Inventory (OLBI) represents a prominent alternative to the tri-factor approach to measuring burnout [13,14]. Consistently with its theoretical structure, the OLBI focuses on two main dimensions: Disengagement and Exhaustion. This model applies to human services professionals and other occupational groups, reflecting its universal applicability [14]. The streamlined two-dimensional focus of the OLBI on Exhaustion and Disengagement allows for a clearer understanding of burnout’s core dynamics, avoiding the ambiguities associated with the broader scope of personal accomplishment. A specific version of the OLBI tailored for nursing students was developed to address this group’s unique stressors and challenges [15]. This adaptation ensures that the measurement of burnout in nursing students is both relevant and precise, capturing the specific aspects of burnout that are most significant in nursing education.

Assessing burnout in nursing students who study in contexts such as India, where the healthcare sector is rapidly expanding and increasingly burdened by both growing population demands and a high prevalence of chronic diseases, becomes critically strategic [16]. The challenges faced by nursing students in India—such as socio-economic pressures, the transitional phase from student to healthcare professional, and cultural expectations—make it strategic to collect information concerning the burnout levels of nursing students [17,18]. In India, nursing students often attend internships in high-stress environments characterized by long hours, understaffing, and high patient loads, typical stressors contributing to burnout [17,18]. Previous research focusing on nursing students in Bangalore, Karnataka, revealed that the socio-cultural context significantly influences burnout levels [19]. Factors such as the high value placed on academic achievement, societal expectations regarding gender roles, and familial pressures exacerbate stress and contribute to burnout among nursing students. Additionally, the healthcare infrastructure in Karnataka, while advanced in some areas, often struggles with resource limitations and high patient-to-nurse ratios, which further intensify the stress experienced by nursing students during their clinical placements [19,20].

Despite the recognized importance of addressing burnout among nursing students in India, there is a notable paucity of studies that specifically assess burnout levels and delineate meaningful groups based on the characteristics of these students [16,17,18]. This gap in research is significant as it hampers the development of targeted interventions that could effectively address and mitigate burnout in this vulnerable group. The ability to identify and understand distinct subgroups among nursing students who might experience burnout differently could lead to more personalized and, thus, more effective preventative strategies and therapeutic approaches. Therefore, the primary aim of this study was to assess the levels of Exhaustion and Disengagement among nursing students from the Bangalore area (south India) using the modified OLBI by Bulfone [15]. This study also aimed to identify distinct patterns in the burnout components assessed using the OLBI relative to the demographic and academic variables of the students.

## 2. Materials and Methods

### 2.1. Design

This study employed a cross-sectional design, following the Strengthening the Reporting of Observational Studies in Epidemiology (STROBE) guidelines to ensure robust and systematic observational data reporting [21]. The sampling procedure was based on convenience, as this study serves as a pilot to provide foundational information for subsequent research.

### 2.2. Context

This study was conducted at Bangalore College of Nursing, an institution recognized for its nursing programs in the Bangalore area (South of India) and comprehensive training facilities. The government of Karnataka recognizes the institution, and the college is approved by both the Karnataka Nursing Council and the Indian Nursing Council. It offers a variety of programs, including a Bachelor of Science in Nursing (BSc Nursing), which extends over a period of four academic years, and a General Nursing and Midwifery (GNM) diploma that spans three years, followed by a six-month internship.

The college boasts well-equipped labs that meet the Indian Nursing Council requirements and experienced faculty specialized in various aspects of healthcare. The educational curriculum imparts necessary clinical skills and enhances students’ knowledge, abilities, and attitudes to thrive in today’s competitive, health-conscious environment. Healthcare in Bangalore is characterized by a blend of traditional practices and cutting-edge medical technology, supported by numerous top-tier hospitals and research institutes. This backdrop is crucial for nursing students, as it exposes them to a broad spectrum of clinical experiences and healthcare challenges, ranging from urban health issues to innovative medical treatments.

### 2.3. Procedure and Ethical Aspects

Initially, after obtaining approval from the institutional review board at Bangalore College of Nursing (prot. 1/Sept/2023), the research team coordinated with the college administration to schedule data collection sessions. Students were recruited through announcements made in class between October and December 2023. Participation was voluntary, and students were informed that their responses would be anonymous and used solely for research purposes. A written informed consent form was provided to all participants, which they were required to read and sign before participating in the study. The data were collected using the OLBI for nursing students [15]. This questionnaire was distributed in paper format during scheduled class times to maximize response rates. Students completed the questionnaires under the supervision of research assistants to ensure that any queries regarding the questions could be addressed immediately. The recruited eligible cohort consisted of roughly 300 nursing students. The estimated time needed to fill out the response form was 8 min.

### 2.4. Eligibility Criteria

Only students actively enrolled in the Bachelor of Science in Nursing (BSc Nursing) program at Bangalore College of Nursing were eligible. Students from all four years of the BSc Nursing program were included to capture a broad spectrum of experiences and stages in their educational journey. Students enrolled in other programs, such as the General Nursing and Midwifery diploma or any short-course certifications offered by the college, were excluded from the study. Students who were enrolled on a part-time basis or had inactive status (e.g., those on academic leave) at the time of the study were not eligible, and any student who did not fully complete and sign the informed consent form was also not eligible.

### 2.5. Measurements

The study employed a range of measurements to gather demographic information and assess burnout among nursing students. Key variables included age, which was recorded as a continuous variable, and studying hours per week. Living arrangements were also documented, noting whether students lived alone, with family, or with other students. Additionally, the student’s academic year was captured, and participants were classified into one of four categories: first year, second year, third year, or fourth year of the BSc Nursing program.

Motivation for choosing a nursing career was another area of focus, with students selecting from options that included altruistic motives such as the desire to help others, practical considerations like job security, personal experiences in healthcare, influence from peers, or pressure from family and friends. As a nominal variable, motivation was developed based on previous literature [22,23,24].

Burnout was assessed using the OLBI nursing student version (OLBI-N), which measures two main dimensions: Exhaustion and Disengagement [15]. Exhaustion reflects the physical and emotional fatigue experienced by the students, while Disengagement measures their mental withdrawal from study-related activities. The validity and reliability of the OLBI-N were previously evaluated using a sample of 476 nursing students. The instrument’s validity was assessed through explorative and confirmative factor analyses. Additionally, to test hypotheses about the relationships between burnout and other psychological constructs, the correlation between burnout as measured by the OLBI-N and academic self-efficacy was examined. The first 7 items measured Exhaustion, while Disengagement was measured by the last 7 items. The minimum score for each domain was 7, while the maximum was 35, as each item was based on a 5-point Likert scale.

### 2.6. Data Analysis

An initial assessment was conducted to evaluate the missing data rates, which were lower than 5%, analyze the missingness pattern, and review each variable’s distribution. For quantitative variables, normality was tested; variables that followed a normal distribution were summarized using means and standard deviations (SD), while those not normally distributed were described using medians and interquartile ranges (IQR). A bivariate analysis was also performed to describe the relationships between scores (Exhaustion and Disengagement) and the other ordinal and quantitative variables (i.e., age, year of the course, weekly studying hours).

To delve deeper into the characteristics of the nursing students—specifically, age, year of the course, weekly studying hours, and the scores for Exhaustion and Disengagement—the t-distributed Stochastic Neighbor Embedding (t-SNE) procedure was employed [25]. A perplexity setting of 30 was chosen for the t-SNE analysis due to the diverse nature of the quantitative variables involved. This choice was favored over other dimensionality reduction techniques, such as Principal Component Analysis (PCA), to better capture the non-linear relationships between variables [25].

The three-dimensional components derived from the t-SNE analysis were visualized in a 3D scatterplot to facilitate an intuitive understanding of the data structure and relationships [25]. Subsequently, these components were utilized in a hierarchical cluster analysis to identify distinct groups within the data using Ward’s method [26]. The selection of the most appropriate cluster solution was guided by silhouette analysis, which evaluated different possible solutions ranging from two to four clusters [27,28]. The interpretability of the results also played a crucial role in this decision.

Once the optimal clusters were identified, descriptive statistics were used to profile each cluster’s characteristics. Differences across clusters were investigated using multiple comparison tests, with the Benjamini–Hochberg procedure applied to adjust for multiple *p*-values, ensuring the findings’ robustness against type I errors [29]. All analytics were performed using R software, version 4.2.2 R [Core Team (2023)], with specific packages including readxl, GGally, ggplot2, stats, tsne, RColorBrewer, and dplyr. The analyses were two-tailed, with a significance level set at 5%.

## 3. Results

### 3.1. Sample Characteristics

The sample characteristics of the study are detailed for the 237 female nursing students who participated (response rate = 79%), as shown in Table 1. The students’ ages ranged from 18 to 28 years, with an average age of 20.65 years (SD = 1.32). Students reported a wide range of weekly study hours from 2 to 100 h, with an average of 31.85 h per week (SD = 17.32). Living arrangements predominantly involved living with other students, which was the case for 92.4% of the participants. A smaller percentage lived alone (2.5%) or with family (5.1%), reflecting the typical living situations for students in this educational context. The sample included students from all four years of the Bachelor of Science in Nursing (BSN) program: 20.7% were first-year students, 29.5% were in their second year, 23.2% were in their third year, and 26.6% were fourth-year students.

The motivations for choosing a nursing career varied among the students, with 48.9% oriented by the possibility of finding work easily, 21.9% motivated by the desire to help others and smaller percentages influenced by positive healthcare experiences or pressure from family and friends.

### 3.2. Exhaustion and Disengagement

Regarding burnout assessments, the internal consistency (Cronbach’s alfa) for OLBI was measured at 0.813, and for Disengagement, it was 0.801. The average scores for burnout dimensions were 25.84 (SD = 4.92) for Exhaustion and 18.86 (SD = 5.00) for Disengagement. Exhaustion ranged from 12 to 35, while Disengagement ranged from 7 to 32.

### 3.3. Bivariate Relationships

**Figure 1:**
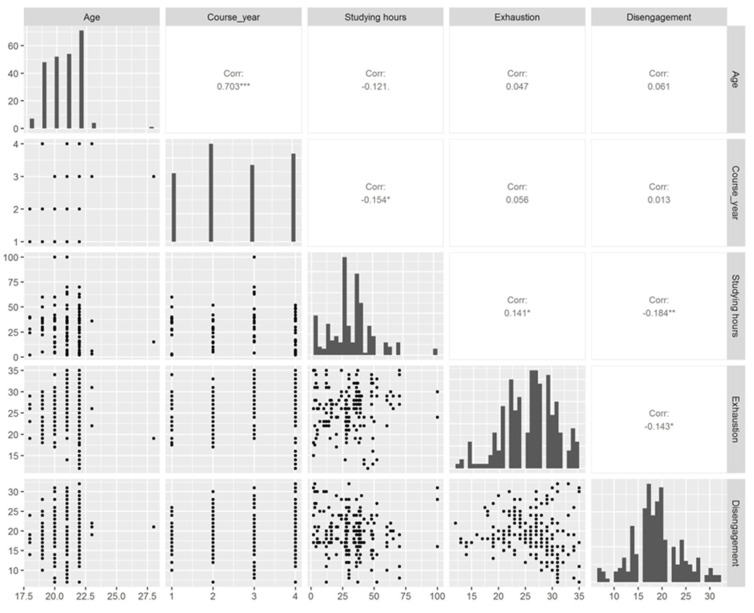
Correlogram. * indicates *p*-values lower than 0.05, ** indicates *p*-values lower than 0.01. *** indicates *p*-values lower than 0.001.

Figure 1 provides a correlogram to show a visual and statistical representation of the bivariate relationships between the variables of interest in the study. In the matrix, the histograms along the diagonal show the distribution of single variables, while the scatter plots and the correlation coefficients in the lower triangle display the relationships between pairs of variables.

Students in higher course years had a higher age (r = 0.703; *p*-value < 0.001) and tended to dedicate fewer weekly hours to studying (r = −0.154; *p*-value = 0.017). Students who dedicated more weekly hours to studying reported a higher Exhaustion score (r = 0.141; *p*-value = 0.031). Both Exhaustion and Disengagement have negative correlations with Course year (r = −0.184, *p*-value = 0.004 for Disengagement and r = −0.143, *p*-value = 0.028 for Exhaustion), suggesting that as students progress in their academic journey, they may experience lower levels of these burnout dimensions.

### 3.4. t-SNE

**Figure 2:**
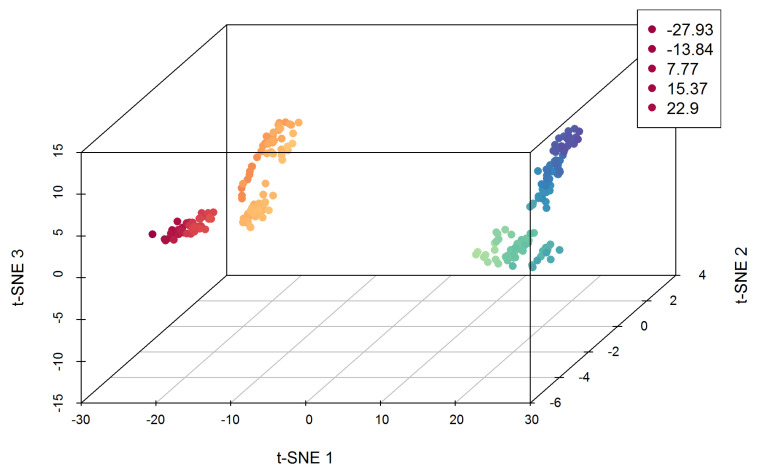
Three-dimensional scatter plot of the t-SNE components.

t-SNE Component 1 (tsne_1) has values ranging from −27.93 to 22.90 and SD of 17.03. t-SNE Component 2 (tsne_2) is narrower than for tsne_1, with minimum and maximum values of approximately −4.20 and 3.75, respectively, and SD = 2.18. t-SNE Component 3 (tsne_3) has a range from −12.10 to 11.05, with SD = 6.78. There is a strong positive correlation (r = 0.793, *p*-value < 0.001) between tsne_1 and tsne_2, while a negative one exists between tsne_1 and tsne_3 (r = −0.339, *p*-value < 0.001) and between tsne_2 and tsne_3 (r = −0.695, *p*-value < 0.001). The 3D scatter plot visualizes the t-SNE components (Figure 2). It shows from two to four distinct patterns, while the data points in 3D space are organized mainly in two parts of the scatterplot.

### 3.5. Hierarchical Clustering Procedure

**Figure 3:**
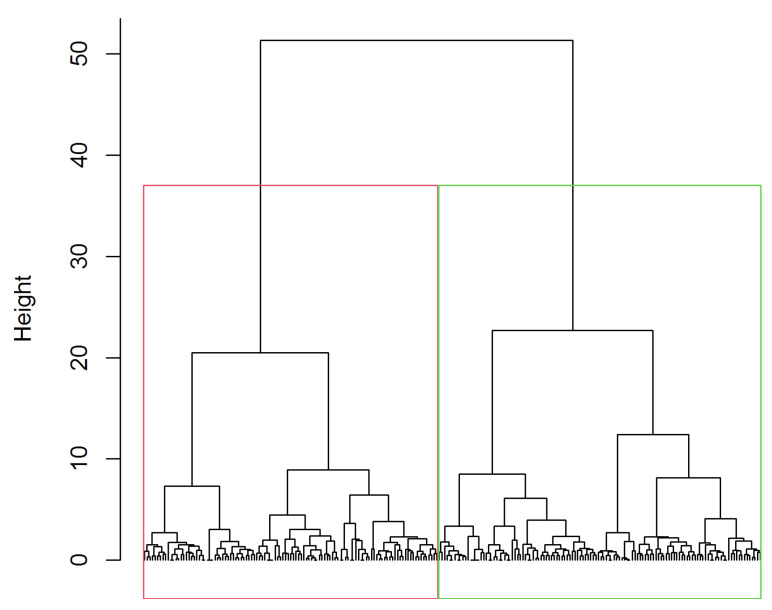
Dendogram and identification of the clusters.

A hierarchical clustering procedure was performed, cutting the dendrogram to extract two, three, and four clusters. The silhouette widths for the solutions with two, three, and four clusters are 0.74, 0.72, and 0.71, respectively. All three values are relatively high, suggesting that the clusters are well separated and distinct from one another in each solution. The two-cluster solution has the highest average silhouette width, which implies that, on average, each object lies closer to the center of its cluster. Therefore, the two-cluster solution offered the most distinct and clearly defined separation of the data into groups (Figure 3), and its solution was used to describe and compare the characteristics of nursing students (see Table 1).

Both clusters had students aged between 18 and 28, and the mean age was closely matched, suggesting that age was not significantly different between the clusters. A significant difference was observed in the weekly studying hours. Cluster 1 students reported a significantly higher mean number of study hours per week (45.56 h) than Cluster 2 students (20.04 h), indicating that the first cluster had a more intensive study schedule (*p*-value < 0.001).

There were no significant differences in living arrangements between the two clusters. Significant differences were observed across the academic years between the clusters. Cluster 1 had a higher percentage of first-year (34.51%) and third-year students (33.63%), while Cluster 2 had more second-year (46.77%) and fourth-year students (31.42%) (*p*-value < 0.001). Motivational factors showed significant variations between the clusters. Cluster 1 had a higher percentage of students motivated by the prospect of finding work easily (67.26%), while Cluster 2 had a higher percentage of students motivated by the desire to help others (29.03%) (*p*-value < 0.001). The internal consistency for the Exhaustion and Disengagement scales, measured by Cronbach’s alpha, was consistent across both clusters, indicating reliable measurements.

Cluster 1 reports a higher mean score compared to Cluster 2 (26.78 ± 5.26 vs. 25.00 ± 4.48; *p*-value = 0.005). Cluster 1 exhibited a lower mean disengagement score of 17.84 (SD = 4.74), whereas Cluster 2′s mean score was 19.78 (SD = 5.08) (*p*-value = 0.002).

Considering the characteristics that distinguish the two clusters, descriptive names that reflect the primary attributes of each group were assigned. Cluster 1 was named “High-Intensity Study Group”, reflecting the significantly higher mean number of study hours per week and the associated higher exhaustion scores. Cluster 2 was labeled “Altruistic Aspirants”, considering the higher percentage of students motivated by the desire to help others suggests a more altruistic drive and the tendency towards higher disengagement scores, which might reflect a sort of altruistic stress driven by a strong desire to help others may find themselves at odds with the reality of their academic and clinical experiences, leading to emotional and cognitive disengagement.

## 4. Discussion

The principal novelty of this study lies in its nuanced exploration of burnout among nursing students, which resulted in being highly prevalent in the educational context in Bangalore, India, utilizing an innovative analytical approach. The study uncovers intricate patterns within the data that traditional methods might overlook by employing t-SNE to manage and simplify the available information [30]. Identifying two distinct clusters, termed “High-Intensity Study Group” and “Altruistic Aspirants”, provides a novel insight into how different motivational drivers and academic pressures relate to the students’ burnout experiences. This approach advances the understanding of burnout in the context of nursing education in India and also showcases the potential of advanced data analysis techniques in revealing the complex interplay between educational demands, personal motivations, and well-being among future healthcare professionals [31,32,33].

This study’s methodology and findings offer a promising avenue for targeted interventions and underscore the significance of considering academic and altruistic dimensions in addressing burnout. Generally, burnout scores are aggregated for the overall group of study participants, which can obscure the nuanced experiences of specific subgroups [8,9]. In this study, the cluster analysis performed by capturing the sample information reduced into simple dimensions using t-SNE allowed for more in-depth insights into the varied experiences and factors contributing to burnout among nursing students. This method helps to highlight the unique stressors faced by different clusters and also enables the development of tailored interventions that address the specific needs and challenges of each subgroup (cluster).

Identifying the “High-Intensity Study Group” suggests interventions that enhance time management and study efficiency, reduce stress through mindfulness and resilience training programs, and offer academic counseling to create a balanced academic and personal life [34]. These students could greatly benefit from learning how to streamline their study habits to reduce Exhaustion without compromising their academic performance. For the “Altruistic Aspirants”, interventions might include sessions that provide a realistic job preview of the nursing profession to align their idealistic expectations with the practical aspects of the job [35]. Mentorship programs could offer emotional support and professional guidance, while structured volunteering opportunities could satisfy their altruistic attitudes in a less pressured context than the academic environment.

The high burnout scores among nursing students, as evidenced in this study, indicate a potentially significant problem within the educational and healthcare systems [36]. High levels of Exhaustion and Disengagement among students are concerning as they may have immediate and long-term consequences on students’ health, educational attainment, and future professional practice [37]. High exhaustion scores could be indicative of chronic stress and overload, which are known to have negative effects on physical and mental health. Over time, this aspect could lead to serious health problems, impair cognitive functioning, and decrease academic performance [37]. Moreover, it raises concerns about the well-being and retention of students within the nursing program, as prolonged stress could lead to higher dropout rates. Disengagement, on the other hand, represents a mental and emotional distancing from one’s studies and future profession [13,14,15]. It could be a protective mechanism against burnout. Still, it may also result in a lack of commitment, decreased performance, and a potential decline in the quality of patient care once these students enter the workforce. High disengagement may also diminish the sense of personal accomplishment and satisfaction that is crucial for a fulfilling career in nursing [38].

The overall high burnout scores signal the need for systemic changes [32,34,35,36,37]. It emphasizes the importance of fostering a supportive learning environment, adjusting academic workload, and providing resources for stress management. Additionally, it underscores the necessity for ongoing monitoring of student well-being and implementing preventive strategies throughout nursing education. The findings also prompt a discussion about the culture within nursing education and the broader healthcare context. It raises questions about societal expectations, the structure of nursing programs, and the support systems in place for nursing students. Addressing these issues is critical for the health and success of individual students, the sustainability of the nursing profession, and the quality of healthcare delivery.

One of the major strengths of this study is its use of t-SNE and hierarchical clustering techniques, which are not commonly applied in traditional burnout research. These methods allowed for the visualization and identification of complex patterns in high-dimensional data that might have been missed with more conventional statistical approaches. This innovative analytical framework provided a deeper understanding of the interplay between various factors influencing burnout. The study’s segmentation of nursing students into two distinct clusters based on their motivational drivers and academic pressures offers a fresh perspective on how these elements correlate with burnout. This nuanced categorization helps tailor interventions more precisely, potentially increasing their effectiveness in preventing burnout. Conducting this research in Bangalore, India, contributes valuable insights into burnout in a non-Western context, helping to fill a gap in the existing literature that predominantly focuses on Western countries. This diversity enhances the study’s applicability and relevance to global health education strategies.

In this study, several limitations warrant acknowledgment. The study’s cross-sectional nature limits its ability to establish causality between the observed factors and burnout. Longitudinal research would be necessary to determine these relationships’ directions and observe how student burnout develops and changes over time. The use of a convenience sample may limit the generalizability of the findings. Students who chose to participate might differ systematically from those who did not, potentially introducing selection bias. The study’s dependence on self-reported measures for data collection, including burnout, academic workload, and motivation, can introduce response biases. Participants might underreport or overreport certain behaviors or feelings, affecting the accuracy of the data. While the study was set in an Indian context, the lack of detailed demographic data (such as socio-economic status, ethnic background, or prior educational experience) may hinder a fully comprehensive understanding of the factors influencing burnout. These variables could significantly impact students’ experiences and outcomes. Additionally, it is important to note that all the recruited nurses were female, which, although common in the Indian nursing context, limits the generalizability of the findings. This gender homogeneity restricts the applicability of the results to male nursing students or more gender-diverse nursing populations, which could potentially exhibit different patterns of burnout and motivational drivers. Another limitation is the monocentric nature of the sampling, as all participants were from a single institution in Bangalore. This could limit the generalizability of the findings to other regions or institutions within India, where the socio-cultural and educational environments may vary significantly. These limitations highlight the need for future research to use more diverse and representative samples, longitudinal designs, and comprehensive demographic data to gain a deeper understanding of the factors influencing burnout among nursing students in India.

## 5. Conclusions

The findings underscore the prevalence of burnout among nursing students and highlight the critical need for targeted interventions designed to address the specific challenges different student groups face. For instance, the “High-Intensity Study Group” could benefit from interventions focused on improving time management and reducing stress, while “Altruistic Aspirants” might find sessions that align their idealistic expectations with the realities of nursing more beneficial. Moreover, the study emphasizes the importance of considering various demographic and personal factors that could influence burnout, such as socio-economic status and ethnic background, which were not detailed in this research. Including only female participants also highlights the need for future studies to incorporate a more diverse group of nursing students to enhance the generalizability of the findings. Ultimately, this research calls for a systemic approach in nursing education and practice to foster a supportive learning environment that addresses the symptoms of burnout and tackles its underlying causes. As the demand for skilled nursing professionals continues to grow globally, ensuring the well-being of nursing students becomes imperative to sustain the workforce and enhance the quality of healthcare delivery.

## Figures and Tables

**Table 1:** Sample characteristics.

		Overall (n = 237)		Cluster 1 (N = 113, 47.68%)		Cluster 2 (N = 124, 53.32%)		*p*
		N	%	N	%	N	%	
Age								
	Years, range: 18–28 (m, SD)	20.65	1.32	20.55	1.27	20.74	1.37	0.261
Studying hours per week								
	Hours, range: 2–100 (m, SD)	31.85	17.32	45.56	13.48	20.04	9.66	<0.001
Live with								
	Alone	6	2.5	4	3.54	2	1.61	0.6922
	Family	12	5.1	6	5.31	6	4.84	
	With other students	219	92.4	103	91.15	116	93.55	
Year of the BSN program								
	First-year	49	20.7	39	34.51	10	8.065	<0.001
	Second year	70	29.5	12	10.62	58	46.77	
	Third year	55	23.2	38	33.63	17	13.71	
	Fourth-year	63	26.6	24	21.24	39	31.42	
Motivation								
	The desire to be of help to others	52	21.9	16	14.16	36	29.03	<0.001
	A positive healthcare experience	16	6.8	3	2.65	13	10.48	
	Oriented by the possibility of finding work easily	116	48.9	76	67.26	40	32.26	
	By some pressure from family and friends	47	19.8	16	14.16	31	25.00	
	Mainly determined by the fact that many of my friends have chosen this course	6	2.5	2	1.77	4	3.23	
Internal consistency								
	Exhaustion (Cronbach’s alfa)	0.813		0.809		0.815		
	Disengagement (Cronbach’s alfa)	0.801		0.8		0.803		
Exhaustion								
	Score, range: 12–35 (m, SD)	25.84	4.92	26.78	5.26	25	4.48	0.005
Disengagement								
	Score, range: 7–32 (m, SD)	18.86	5	17.84	4.74	19.78	5.08	0.002

Legend: m = mean; SD = standard deviation. Note: Inferential comparisons were corrected by following the Benjamini–Hochberg procedure.

## Data Availability

The data that support the findings of this study are available from the corresponding author, RC, upon reasonable request. The data are not publicly available due to privacy and ethical restrictions.

## Public Involvement Statement

No public involvement in any aspect of this research.

## Guidelines and Standards Statement

This manuscript was drafted against the Strengthening the Reporting of Observational Studies in Epidemiology (STROBE) guidelines.
